# Working donkey welfare assessment and owner survey in Meru County, Kenya

**DOI:** 10.1017/awf.2025.10031

**Published:** 2025-08-22

**Authors:** Martha Mellish, Jason Stull

**Affiliations:** Department of Health Management, Atlantic Veterinary College, University of Prince Edward Island, PE, Canada C0A 1T0

**Keywords:** Animal welfare, body condition, cart vs pack, hobbling injuries, SEBWAT, working donkey

## Abstract

There are significant welfare concerns regarding the plight of working donkeys (*Equus asinus*) in developing countries. To-date, however, there has been limited work assessing the welfare of donkeys in many parts of Africa, including Kenya. This study aimed to characterise the unique welfare concerns of working donkeys in Meru County, Kenya. Baseline information was gathered, concerning challenges with feeding, working conditions and disease faced by owners and drivers with differences between pack and cart donkeys investigated. To this end, 102 donkeys underwent evaluation using a Standardised Equine Based Welfare Assessment Tool (SEBWAT) and 58 owners were surveyed. Important welfare concerns, including low body condition scores (BCS) (median [IQR] 2 [1.5, 2.5 out of 5]), hobbling (81/102; 79%) and mutilation wounds (49/102; 48%) were identified in all donkeys. The following categories registered significant physical differences between cart and pack donkeys: signalment (cart 100% male, pack 21% male); BCS (median cart 2.0, pack 1.5); and presence of skin wounds on the neck (cart 30%, pack 0%). Behaviour was assessed with differences noted in chin contact avoidance (cart 56%, pack 97%), tail tuck presence (cart 46%, pack 97%), number of donkeys owned (median cart 2, pack 1), reported administration of de-worming medication by owners (cart 95%, pack 17%), and occurrence of reported illness (cart 81%, pack 38%). This initial survey addresses welfare concerns related to the Meru County donkey population and will serve as a useful benchmark for future assessments as well as targeted interventions, including the introduction of modified carts to the region.

## Introduction

The donkey (*Equus asinus*) population of Kenya was surveyed as being 1.1 million in 2019 (The Brooke 2019). Most of these are working donkeys, which play an important role in Kenyan farms and households. Donkeys are present in most smallholder farms, with 93% of rural households reporting at least one donkey on their farm (Carder *et al.* 2019). Half of the households that have donkeys use them for domestic work, with the primary chore being carrying water. As it may be difficult to gain access to water with a motorised vehicle due to lack of roads and steep terrain, water for household use oftens ends up being transported by cart or packed onto the back of donkeys. Such transportation can also provide income through fees for donkeys moving materials, such as farm produce, water and firewood and helping with land cultivation (Carder *et al.* 2019). A 2019 survey (Carder *et al.* 2019) saw 67% of respondents note a noticeable decrease in food availability following loss of a donkey.

The important roles played by these working equids in households does not necessarily translate into their welfare being prioritised (Carder *et al.* 2019; Haddy *et al.* 2020). While research into the welfare of working donkeys has historically been limited (Reix *et al.* 2014; Upjohn *et al.* 2014; Ali *et al.* 2016; Geiger *et al.* 2021), there have been a spate of recent studies looking into the benefits of donkeys in Kenyan production systems, the challenges they face, and methods for assessing their welfare (Gichure *et al.* 2020; Geiger *et al.* 2021). Analysis of interventions to improve welfare have also been described (Leeb *et al.* 2003; Gichure & Olayide 2024). A paucity of regulations are in place at the governmental level of low- to middle-income countries covering the regulation of working conditions for donkeys, such as load weights, food and water provision and appropriate saddling or harnessing (Davis 2019; Bukhari & Parkes 2023). While most areas of Kenya use donkeys for manual labour, in certain regions donkey meat is consumed (Gichure *et al.* 2020; Rickards & Toribio 2021) .

Ongoing export of donkey skins to Asian countries is seen, for use in a traditional Chinese medicine, known as *ejiao* (Carder *et al.* 2019; Gichure *et al.* 2020; Goodrum *et al.* 2022) and the past decade has seen a marked impact on the donkey population in Kenya. The slaughter of animals for their skins has decreased from a reported 1.8 million in 2009 (Karanja-Lumumba *et al.* 2019) to the most recent population survey of 1.1 million in 2019 (The Brooke 2019). The consequences of donkey slaughter, including donkey theft, poor donkey welfare, decimation of the population of these valuable income earners and essential parts of household work saw a public outcry and lead to the closure of Kenyan slaughterhouses in 2020 and in 2023 to an African Union moratorium on donkey skin trade (Cathcart 2024). Cross-border movement of donkeys for the purposes of export continues to be a source of infectious disease and facilitates thefts of donkeys in Kenya which sees an increase in their market value (Waters 2019; Cathcart 2024).

The low number of welfare assessments that have been carried out to-date, suggests that donkeys in Kenya are exposed to poor treatment, including inconsistent, low-quality feed, sporadic watering, ill-fitted and designed harnesses, and mistreatment such as whipping (Davis 2019). These findings vary relative to geographic location and resultant cultural and environmental differences in terms of donkey care (Geiger *et al.* 2021). A donkey welfare survey (Leeb *et al.* 2003) performed by the Kenyan Society for the Protection and Care of Animals (KSPCA) in the Lake Victoria region of Kenya noted an average or above average BCS, decreased leg lesions, increased use of head collars and less overgrown hooves in areas previously visited by the KSCPA. Broader health evaluations of working equids in developing countries such as Kenya gave rise to less favourable findings, including increased rates of parasitic burdens, low body condition, wounds, respiratory disease, lameness and dental problems (Burn *et al.* 2010; Geiger & Hovorka 2015; Ali *et al.* 2016). In certain locations, donkeys may be valued less than other livestock (Haddy *et al.* 2020; Geiger *et al.* 2021; Grace *et al.* 2022) which translates as provision of comparatively lower amounts of food and water. Allied to this is the fact that donkeys typically show greater tolerance of drought conditions, can endure harsh weather and often prevail despite lower levels of care compared to other livestock (The Brooke 2019).

The aim of this study was to characterise the specific welfare concerns facing working donkeys in Meru County, Kenya and gather baseline information regarding the challenges related to feeding, workload and disease. To our knowledge, a welfare assessment of this type in this region has not been previously carried out. A further aim of the welfare evaluation was to determine whether there were any differences in the welfare concerns faced by pack and cart donkeys.

## Materials and methods

### Ethical approval

A cross-sectional survey was performed, including a survey of human participants and a welfare assessment of their donkeys. As an incentive for participating, respondents were given de-wormers for administering to their donkeys. Study approval came from the University of Prince Edward Island’s Animal Care Committee (donkey assessment, Protocol # 22-040) and the Research Ethics Board (human survey participants, File # 6011853).

### Study area and environment

**Figure 1.**
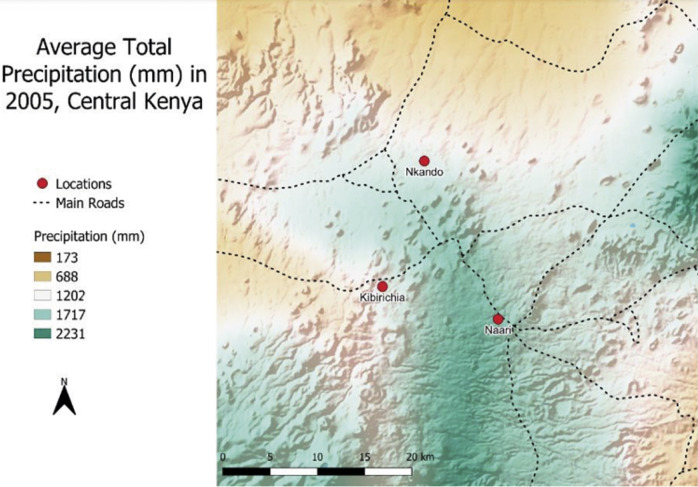
Average total precipitation (mm) in Central Kenya in 2005. Map illustrates rainfall in arid areas (Nkando) where donkeys carry packs and areas of higher rainfall (Naari, Kibirichia) where donkeys traditionally pull carts. Photo credit: Nolan Kressin.

Welfare assessments and owner surveys were carried out in Meru County, Kenya (0^°^N, 37.8^°^E) in January 2023. Meru County covers an area of approximately 7,000 km^2^ with an estimated human population of 1.4 million (Meru 2018). Exact donkey numbers in the county are difficult to gauge since there has been no government census or previous counts. Pack and cart donkeys were surveyed in the specific regions they would typically be found in the county, i.e. in the arid, northern areas of the county (Nkando) donkeys being used to pack water predominate, whereas in those areas with increased rainfall, such as Naari and Kibirichia, donkeys pull carts (Figure 1) (Meru 2018). Population-wise, lower numbers reside in semi-arid areas where the pack donkeys are used (152 people per km^2^) compared to the more populous fertile lands where cart donkeys are used (456 people per km^2^) (Meru 2018). Recruitment was based on a volunteer sample of donkey owners with notification delivered verbally through local donkey associations.

### Animal-based measures

#### Welfare assessment

The Standardised Equine Based Welfare Assessment Tool (SEBWAT) has undergone repeated use by non-governmental organisations in several low- to middle-income countries (Sommerville *et al.* 2018) and was deployed here to evaluate working donkeys. It is a tool that involves 40 animal-based measures capturing general health, behaviour, body lesions, and body condition score (BCS) on a five-point scale (Table 1). Behaviour was observed, including the response to observer approach, general attitude, as well as painful or avoidance reactions from chin contact, tail tuck, and spinal palpation (Table 2). Body lesions were evaluated via visual observation with the donkey not wearing a pack or harness. The body areas were divided into head/ears, neck, withers/spine, hindquarters, tail, breast/shoulder, forelimbs, ribs/flank, girth/belly, genital/rectal and hindlimbs. Lesion severity was evaluated in terms of depth (superficial or healed lesion, extending through skin into subcutaneous layers or deep enough to show muscle tendon or bone). If more than one lesion was present in a specific body area, the most severe lesion was recorded with lesion size estimated using a lesion-measuring template. This was carried out in accordance with the SEBWAT guidelines (Sommerville 2018). Practice-induced conditions, including mutilations, firing (burning the skin with hot metal), and hobbling (tying ropes around the animals’ limbs) were assessed. A gait evaluation took place which consisted of assessing the donkey moving freely or being driven by the handler for at least six paces towards and away from the observer. Stride regularity, limping and the ability to weight bear underwent visual evaluation (Sommerville 2018). Lower limb swelling, as well as hoof and frog health were assessed via palpation and picking up at least one of the donkey’s feet (Sommerville 2018).

**Table 1.** Description of Body Condition Scoring factors used for scoring donkeys (*Equus asinus*) in the Standardised Equine Welfare Based Assessment Tool evaluations (Sommerville *et al.* 2018) and deployed in study of donkeys in Meru County, Kenya

		Body Area					
Score	Description	Neck	Shoulder	Spine	Ribs	Pelvis (viewed from behind)	Tailhead
1	Very Thin	Concave	Point of shoulder prominent	Prominent	Prominent	Concave Hooks and pins prominent	Prominent
2	Thin	Concave or straight	Point of shoulder visible	Visible	Visible	Flat Hooks and pins visible	Visible
3	Medium	Straight Joins the body smoothly	Point of shoulder not clearly visible Joins the body smoothly	Slightly visible at the withers Smooth elsewhere	Not visible	Well-filled	Slight visible, but well filled and joins the rump smoothly
4	Fat	Slightly convex	Some fat accumulation behind the shoulder	Slight ‘gutter’	Some fat accumulation	Well rounded or slightly ‘heart shaped’	Some fat accumulation
5	Very Fat	Distinctly convex	Fat accumulation behind shoulder clearly visible	Fat accumulation on either side of spine with a distinct ‘gutter’	Fat accumulation clearly visible	Distinctly rounded (clearly heart shaped)	Fat accumulation clearly visible Distinctly convex

**Table 2.** Behaviour assessment criteria adopted from Sommerville *et al.* (2018) used in Standardised Equine Welfare Based Assessment Tool evaluations and deployed in study of donkeys (*Equus asinus*) in Meru County, Kenya

Parameter	Score	Description	Scoring Criteria
Observer approach (Sommerville *et al.* 2018)	0	Positive	Equid is not afraid of the approaching observer, and is alert, friendly or relaxed, but not nervous or apathetic
	1	Negative non-reactive	Equid is apathetic, dull or non-responsive, and has no interest in the approaching observer
	2	Negative reactive	Equid is anxious, frightened or aggressive in response to the approaching observer
Chin contact ()	0	Accepts contact	Equid calmly allow chin to be touched
	1	Avoid contact	Equid withdraws the head when contact with the chin is made, or as the hand is approaching the chin
Tail tuck (Sommerville *et al.* 2018)	0	No tail tuck	Equid does not show any sign of tail tuck whilst assessor is walking towards or around the hindquarters
	1	Tail tuck	Equine tucks the tail between the hind limbs, clamps down the tail and/or tucks in or tenses the hindquarters at any time whilst you are walking towards or around the hindquarters
General attitude)	0	Positive	Equid is not afraid, and is alert, friendly or relaxed but not nervous or apathetic throughout the majority of the assessment
	1	Negative non-reactive	Equid is apathetic, dull or non-responsive throughout the majority of the assessment
	2	Negative reactive	Equid is anxious, frightened or aggressive throughout the majority of the assessment
Spinal contact (Sommerville *et al.* 2018)	0	No reaction	Equid shows no clear reaction when contact with the spine is made
	1	Reaction	Equid shows visible tensing of the muscles of the back or neck, flinching of the part of the spine being touched, or clear movement of parts of the body other than the area being touched when contact with the spine is made

Assessments took place at donkeys’ respective farms or at a central location within the target area. Donkeys that pulled carts two-abreast were defined as cart donkeys while donkeys carrying water or goods strapped to their back with rope were pack donkeys and both were included in the study. All foals (seven) were excluded from the study. Data collection was carried out by the same two observers throughout, one of which (MAM) is an equine veterinarian the other an assistant familiar with donkey husbandry. The owner or person in charge of the donkey carried out the restraint for the exam while a translator was on-hand. For each individual donkey, both observers discussed the parameters before coming to an agreement on the value ascribed. Aggression or avoidance behaviour during the initial approach meant physical contact with the donkey was avoided with visual-only aspects of the SEBWAT undertaken. At the conclusion of the exam an oral de-wormer was administered and a tape used to estimate the donkey’s weight.

### Owner survey

One donkey owner per household was interviewed prior to the welfare exam, either in a communal area where owners gathered or at the individual’s farm. If multiple people accompanied the donkey, one of them was asked to volunteer to take the survey. Either MAM or some senior veterinary students under MAM’s direct supervision asked multiple-choice, closed and open-ended questions with the help of a translator fluent in the local dialects (kikuyu, kimeru and/or Swahili). Since these interactions were conducted verbally and in person the ability to read and write was not required. A signature from the owner was taken immediately following verbal consent for the survey, and notes were taken on questionnaires. The two-page questionnaire covered topics that included owner demographics, farm husbandry provided to the donkey, working conditions, preventative healthcare knowledge, donkey illness history, perceived challenges with working donkeys, number of donkey(s) owned by the household, as well as how the donkey was acquired. Preventative healthcare practices, such as vaccination frequency and use of de-wormers were covered via closed-ended questions with multiple-choice options depending on the initial answer (see entire questionnaire in the Supplementary material).

### Statistical analysis

Data were entered into an Excel® database (Microsoft Corp, Redmond, WA, USA) before being analysed using Intercooled Stata version 17.0 (Stata Corp, College Station, TX, USA). Continuous variables were assessed for normality using the Shapiro-Wilk test. Descriptive statistics were computed for all variables and stratified by type of work (transport goods by cart, transport goods by pack). Blank and ‘don’t know’ responses were excluded from analyses. Statistical associations were assessed between type of work and each variable. Associations with categorical variables were assessed using Pearson *χ* ^2^ test or Fisher’s Exact test. If any expected cell value was ≤ 1 or 20% or more expected cell values were ≤ 5, the Fisher’s Exact test was used; otherwise, Pearson *χ* ^2^ test was used. Associations with continuous variables were assessed using the two-sample Wilcoxon rank-sum (Mann-Whitney *U*) test (when non-normally distributed variable) and two-sample *t*-test (when normally distributed variable). *P*-values ≤ 0.05 were considered statistically significant.

## Results

Of the 102 working donkeys surveyed, 68 pulled carts and were evaluated in Naari and Kibirichia while the 34 pack donkeys were assessed in Nkando.

### Animal-based measures

Cart donkeys were exclusively male, which contrasted with the mixed sexes typically seen in the pack donkeys (*P* < 0.001; Table 3). The donkeys’ bodyweights (with measurements taken via weight tape) were similar for two groups (*P* = 0.06). The cart donkeys however had a significantly higher BCS compared to the pack donkeys (median 2.0 vs 1.5; *P* < 0.001). This difference became even more pronounced when restricted to male donkeys (median cart 2.0 vs pack 1.0; *P* < 0.001).

**Table 3.** Results of Standardised Equine Welfare Based Assessment Tool (SEBWAT) performed on 102 working donkeys (*Equus asinus*) in Meru County, Kenya

Question (n)	Overall (%)	Transport goods by cart (TGC) (%)	Transport goods by pack (TGP) (%)	Comparison of TGC and TGP (*P*-value)
*Sex (TGC = 68; TGP = 33)*				< 0.001^a^
Male	75 (74%)	68 (100%)	7 (21%)	
Female	26 (26%)	0	26 (79%)	
*Age (TGC = 66; TGP = 34)*				0.45^a^
Less than 3.5 years	11 (11%)	5 (8%)	6 (18%)	
3.5–7.9 years	29 (29%)	21 (32%)	8 (24%)	
8.0–12.0 years	29 (29%)	19 (29%)	10 (29%)	
Over 12 years	31 (31%)	21 (32%)	10 (29%)	
*Height; hands or 4 inch increments (TGC = 64; TGP = 33)*				0.0021^b^
Median (IQR)	10.1 (10.0, 10.2)	10.1 (9.3, 1–0.1)	10.1 (9.3, 10.1)	
*Weight via tape; kg (TGC = 63; TGP = 33)*				0.06^f^
Mean (± SD)	137.8 (± 20.0)	140.5 (± 19.5)	133.6 (± 22.7)	
*Body Condition (TGC = 66; TGP = 32)^d^*				< 0.001^b^
Median (IQR)	2 (1.5, 2.5)	2 (2.0, 2.5)	1.5 (1.0, 2.0)	
Males only Median (IQR)	2.0 (2.0, 2.5)	2.0 (2.0, 2.5)	1.0 (1.0, 2.0)	< 0.001^b^
*Observer Approach (TGC = 68; TGP = 34)^e^*				0.72^c^
Positive	5 (5%)	4 (6%)	1 (3%)	
Non-reactive	63 (62%)	43 (63%)	20 (59%)	
Negative reactive	34 (33%)	21 (31%)	13 (38%)	
*Chin Contact (head shy) (TGC = 68; TGP = 34)^e^*				< 0.001^a^
Accepts chin contact	31 (30%)	30 (44%)	1 (3%)	
Avoids chin contact	71 (70%)	38 (56%)	33 (97%)	
*Tail Tuck (TGC = 68; TGP = 34)^e^*				< 0.001^a^
No tail tuck	38 (37%)	37 (54%)	1 (3%)	
Tail tuck present	64 (63%)	31 (46%)	33 (97%)	
*General Attitude (TGC = 68; TGP = 34)^e^*				0.082^a^
Positive	14 (14%)	13 (19%)	1 (3%)	
Non-reactive	69 (68%)	43 (63%)	26 (76%)	
Negative reactive	19 (19%)	12 (18%)	7 (21%)	
*Spinal Contact (TGC = 66; TGP = 34)^e^*				0.76^c^
No reaction	87 (87%)	58 (88%)	29 (85%)	
Reaction	13 (13%)	8 (12%)	5 (15%)	
*Neck skin lesion/Severity (TGC = 66; TGP = 34)*				< 0.001^c^
No lesion	80 (80%)	46 (70%)	34 (100%)	
Healed lesion	18 (18%)	18 (27%)	0	
Non-healed lesion	2 (2%)	2 (3%)	0	
*Mutilations: Tail, Ear, Muzzle (TGC = 68; TGP = 34)*				0.48^a^
No mutilation	53 (52%)	37 (54%)	16 (47%)	
Healed mutilation	49 (48%)	31 (46%)	18 (53%)	
*Hobbling lesion: Body areas (TGC = 68; TGP = 34)*				0.10^a^
No lesion	21 (21%)	14 (21%)	7 (21%)	
Healed lesion	13 (13%)	12 (18%)	1 (3%)	
Non-healed lesion	68 (67%)	42 (62%)	26 (76%)	
*Gait (TGC = 68; TGP = 34)*				0.70^c^
No lameness at walk	98 (96%)	64 (94%)	34 (100%)	
Moderate compromise of gait at walk	1 (1%)	1 (1%)	0	
Severe compromise of gait at walk	3 (3%)	3 (4%)	0	

Columns may not sum to 100% due to rounding.

a Pearson Chi squared test.

b Two-sample Wilcoxon rank-sum (Mann–Whitney *U*) test.

c Fisher’s Exact test.

d See Table 1 for details on body condition scoring.

e See Table 2 for details on scoring.

f Two-sample *t*-test with unequal variances.

No statistical differences were noted in response to observer approach, general attitude or spinal contact between the pull cart and pack water groups (all *P* > 0.08). Chin contact avoidance and the presence of a tail tuck when walking behind the donkeys were observed more frequently in pack (each 97%) compared to cart donkeys (56, 46%; *P* < 0.001).

**Figure 2.**
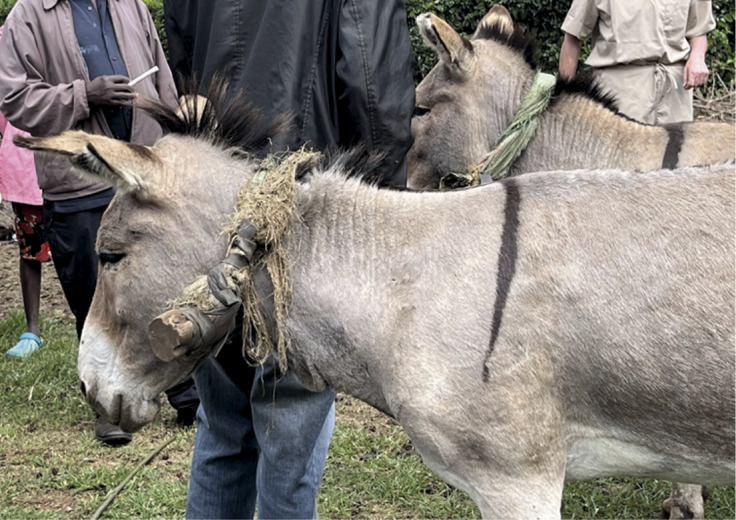
Ear notching, skin hypertrophy on the neck and commonly used harness for cart donkeys assessed in Meru County, Kenya.

Ear and muzzle mutilations were frequently observed in both groups with donkeys showing healed or ulcerated, owner-induced lacerations (cart: 46; pack: 53%; Table 3, Figure 2). Firing (deliberate skin lesions created by heated metal) were present in both groups, with hobbling injuries seen in 79–80% of pack and cart donkeys. Six percent of cart donkeys were observably lame at the walk while in pack donkeys, no observable gait deficits at the walk were seen.

**Figure 3.**
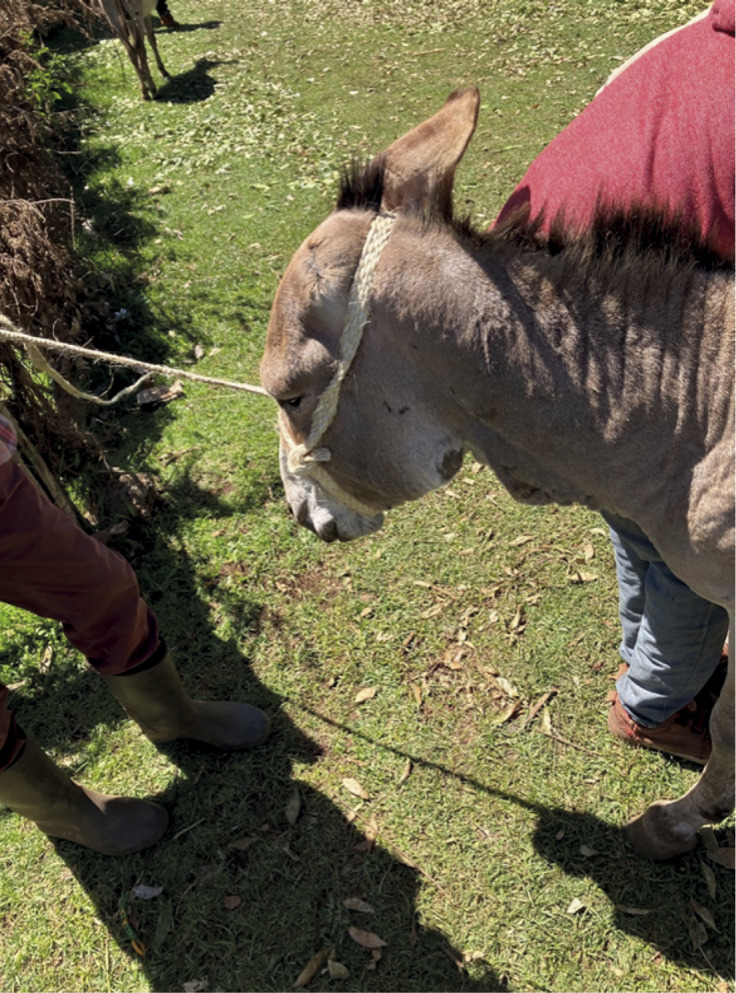
Hypertrophied skin on ventral neck and loss of hair on jaw from harnessing. This donkey had an ear amputation performed by a veterinary technician due to a non-healing injury in Meru County, Kenya.

Skin lesions, such as healed or ulcerated skin on the neck, were observed more frequently on cart compared to pack donkeys (Figure 3) (30, 0%; *P* < 0.001). Occurrence of skin lesions on other body parts were low (for full results of the SEBWAT, see Supplemental material).

### Owner survey results

Pack donkey owners frequently reported (81%) the challenge of having enough feed for their donkeys or an overall lack of available food. A greater proportion of health and safety concerns were reported for cart donkey owners compared to pack owners (Health: 62 vs 41%, Safety: 35 vs 22%, respectively).

Most cart donkeys were reportedly worked 1–3 days a week, with pack donkeys mostly being reported as working 4–7 days a week (Table 4; *P* < 0.001). Owners of cart donkeys reported that their animals pulled significantly heavier weights than those carried by pack donkeys (median 500 vs 80 kg; *P* < 0.001).

**Table 4.** Kenyan donkey (*Equus asinus*) owner survey questions (total of 58 owners) with differing showing responses from donkeys that transport goods by cart and donkeys that transport goods by pack. Seven questions are described out of a ten-question survey

Question (n)	Overall (%)	Transport goods by cart (%)	Transport good by pack (%)	Comparison of TGC and TGP (*P*-value)
*How many donkeys do you or your family own or drive? (TGC = 28; TGP = 30)*				
Median (IQR)	2 (1, 2)	2 (2, 2)	1 (1, 2)	< 0.001^a^
*How do you get the donkey feed? (TGC = 28; TGP = 25)*				NP
Harvest yourself	14 (26%)	9 (32%)	5 (20%)	
Buy	3 (6%)	1 (4%)	2 (8%)	
Graze on common property	51 (96%)	26 (93%)	25 (100%)	
*Do you de-worm your donkey? (TGC = 20; TGP = 30)*				< 0.001^c^
No	26 (52%)	1 (5%)	25 (83%)	
Yes	24 (48%)	19 (95%)	5 (17%)	
*How many days a week do the donkeys work? (TGC = 21; TGP = 29)*				< 0.001^b^
1–3 days a week	20 (40%)	16 (76%)	4 (14%)	
4–7 days a week	27 (54%)	5 (24%)	22 (76%)	
Not working (too young, too old, lame)	3 (6%)	0	3 (10%)	
*How much weight do the donkeys carry on average? (TGC = 26; TGP = 28)*				
Median (IQR)	120 (80, 500)	500 (350, 500)	80 (80, 85)	< 0.001^a^
*Have you owned a donkey that has been sick? (TGC = 27; TGP = 24)*				0.001^c^
Yes	31 (61%)	22 (81%)	9 (38%)	
No	20 (39%)	5 (19%)	15 (63%)	
*What are the challenges you face with your donkey? (TGC = 26; TGP = 27)*				NP
feeding (food+water+fodder+feed)	26 (49%)	4 (15%)	22 (81%)	
Behaviour (slow, behaviour, tired, rest, refuse)	9 (17%)	7 (27%)	2 (7%)	
Health (de-worming, worms, disease, wounds, sick, disease, foot, limp, pneumonia, tsetse fly)	27 (51%)	16 (62%)	11 (41%)	
Safety (falling, slaughter, predators, tether, lost, traffic, park, cut)	15 (28%)	9 (35%)	6 (22%)	

a Two-sample Wilcoxon rank-sum (Mann–Whitney *U* test).

b Fisher’s Exact test.

c Pearson Chi squared test.

NP – not performed.

Differences in practices related to de-worming were seen, with significantly higher rates reported in cart donkeys compared to pack (95, 17%; *P* < 0.001). Lower rates of illness were reported in pack compared to cart donkeys (38, 81%; *P* < 0.001).

## Discussion

Welfare concerns identified in the working donkeys of Meru County included thin and very thin BCS values as well as negative or non-reactive responses to observer approach and general attitude. Skin lesions likely due to work type and practice-induced conditions and mutilations were commonplace. Challenges with feeding donkeys, especially pack donkeys around Nkando were reported. The donkeys’ workload was consistent with the type of work performed: pack donkeys tended to haul water daily for household use, whereas cart donkeys hauled goods for market and farm use 1–3 days a week. Higher rates of de-worming and reported illnesses were seen in the cart donkeys.

Cultural barriers specific to the region likely contribute to donkey welfare challenges in Kenya since donkeys tend to more often be rented than owned. There are also deep-rooted social issues that contribute to a lack of social cohesion between communities and a lack of empathy for working equids that negatively impact welfare (Pritchard *et al.* 2018). Specific regions in Kenya show varying combinations of these issues.

The survey results encapsulated the insecurity around food availability both for humans and animals in Meru County while the animal-based measures illustrate what is deemed typical in terms of the physical harm inflicted upon animals in the form of work-related skin lesions and for identification. Also readily apparent from the survey was the donkeys’ undeniable importance both in terms of income generation and household chores.

### Sex and BCS

The observed difference in sexes between pack (mixed sexes) and cart (all males) donkeys was likely due to a number of factors. Donkeys that pull carts tend to be bought, meaning that a male donkey (a jack) can be attained preferentially over a female (a jenny) when it comes to pulling. It is also a common belief locally that only jacks can pull carts. The perceived increased strength of males and owner preferences for males for work-related tasks were also seen in working donkey surveys in Botswana and Ethiopia (Geiger & Hovorka 2015; Geiger 2023). This is also illustrated in studies from Pakistan, Ethiopia and Mali (McLean *et al.* 2012; Reix *et al.* 2014; Ali *et al.* 2016; Geiger *et al.* 2021). In this study, the gender of the donkey owner was not unfortunately recorded since, traditionally, men use donkeys for income generation while women employ them for household tasks. The type of work the donkey performs and the household member that drives the donkey shapes the human-donkey relationship and the related welfare of the donkey (Geiger 2023). The gender distribution of owners may also influence the sex of the donkey owned. Subjectively, MAM noted that donkey cart drivers were all males whereas for pack donkeys mixed gender was observed. This is reflected in the sex distribution of the donkeys. Displays of sexual behaviour between jacks and jennies may also govern the sex distribution. Normal sexual behaviour when sexes are mixed includes jacks vocalising and engaging in the aggressive pursuit of jennies to interact with them (Henry *et al.* 1998). This behaviour is loud and potentially disruptive to owners.

The donkeys in this study had a thin (score of 2) and very thin (score of 1) BCS. This is comparable to the published mean BCS of working mules/donkeys studied in Ethiopia (mean BCS of 2.8), Botswana (mean BCS of 2), Pakistan (mean BCS of < 2) and Mali (mean BCS of 2.3) (McLean *et al.* 2012; Upjohn *et al.* 2014; Reix *et al.* 2014; Ali *et al.* 2016, 2019). The higher BCS of donkeys that pull carts may be due to increased availability of food in the specific geographical areas cart donkeys typically inhabit. Both cart and pack donkey owners report that donkeys are not commonly supplied with food. All donkeys are tethered to allow grazing or herded to a common land area where they can graze. As a result, their food supply is highly dependent upon the weather and edible plant availability. The lower average annual rainfall in pack donkey areas results in a visible difference in available forage which may cause the lower BCS for pack donkeys. In Mexico, working equids in the most arid area showed significantly worse welfare compared to donkeys in more humid areas. The authors also suggested that the poor-quality forage resulting from the environmental conditions may also contribute to the lower BCS seen in this area (Haddy *et al.* 2020). This study also showed differences in BCS according to work type, with pack equids having a thinner BCS than riding equids (Haddy *et al.* 2020). A correlation between lameness and a low BCS has been found. Pain and a reduced appetite as well as the increased energy requirement of moving when in pain may also explain this association, as well as malnutrition (Reix *et al.* 2014). The number of lame donkeys in this study was relatively low at 6%. Pain in limbs may be less likely to be a cause of a low BCS with these findings. Food insecurity is reflected in the owner surveys, with 81% of owners in the pack donkey areas reporting feeding to be a challenge. This compares with 15% of cart donkey owners listing feeding as a challenge. When households are struggling to meet basic needs for humans, the ability to address animal health issues is limited (Upjohn *et al.* 2014; Geiger & Hovorka 2015).

### Behaviour comparison between cart and pack donkeys

The frequently observed lack of response to observer approach noted here (63/102; 62.7%) showed similarities with the Burn *et al.* (2010) study utilising the SEBWAT to evaluate 5,481 donkeys in developing countries, including Kenya, in which no response was seen in 64.6% of donkeys and avoidance aggression in 26.2%. A study of 100 working donkeys in Botswana had a 69% occurrence of dull demeanour or avoidance behaviour (Geiger & Hovorka 2015) which was echoed in the results seen in working donkeys in brick kilns in Pakistan and working mules/donkeys and horses in Mexico (Haddy *et al.* 2020; Bukhari & Parkes 2023). In the current study, overall, 18.6% showed negative reactive behaviour that consisted of jumping up and down on the front limbs, turning the hindquarters towards the observer and kicking. These were often younger donkeys, and the owners predicted the negative reactive behaviour that would likely occur with interactions. Younger animals with a higher BCS have been noted as having a greater likelihood of displaying aggression towards their handler (Burn *et al.* 2010). In general, however, a higher BCS is associated with an improved general attitude toward humans (Bukhari & Parkes 2023).

Unresponsiveness of working donkeys has been linked with poor health, such as a low BCS and abnormal mucous membrane colour (Burn *et al.* 2010). The health status of the donkey combined with their previous experience of human interactions impact their reaction to a novel human. Such a high rate of uninterested and aggressive behaviour could indicate that the donkeys do not have regular positive human interactions. It has been seen in working mules that aggressive interactions can be initiated by inappropriate, physically rough handling (Ali *et al.* 2019). A lack of knowledge from donkey owners regarding normal donkey behaviour (Davis 2019) and limited use of positive training methods, especially in stressful situations (such as a group of donkeys together), may result in donkeys both actively and passively avoiding human interaction (Burn *et al.* 2010). Low levels of knowledge as regards appropriate handling skills can result in donkeys being beaten, inducing fear and stress (Bukhari & Parkes 2023). MAM observed the most physical resistance from donkeys occurring during initial restraint. As the donkeys were either led with a rope around the leg or were loose, restraining the donkey for the welfare evaluation necessitated control of the donkey’s head by a headcollar. The main method of donkey restraint entailed holding and/or twisting their ears, resulting in severe avoidance by the donkeys regarding touching of their ears (also known as ‘ear-shy’). Encouragement, as well as MAM and the research team demonstrating deliberate and considerate head restraint, placement of a headcollar and desisting from using the ears often gave rise to adequate restraint requiring reduced conflict. Owners were often observed prodding and physically striking their donkeys during herding them to the central meeting points. It would be helpful, moving forward, to carry out surveys of handling methods to establish common training techniques. One limitation of the behaviour evaluation was that time constraints meant the majority of donkeys had to be observed at central meeting points with only a minor subset evaluated at their home farm. Donkeys are territorial (The Donkey Sanctuary 2021), and a novel location can significantly impact on their behaviour. It would have been preferable to have evaluated donkeys at their home farm.

MAM observed both cart and pack donkeys being poked, prodded and hit with sticks on their sides and hindquarters to direct them while working. This can lead to the expectation of pain when a human or handler walks behind the donkey (McLean *et al.* 2012). Tail tucking when walking around the hindquarters was noted more frequently in pack compared to cart donkeys. This may reflect an increased use of negative reinforcement using sticks with pack donkeys compared to cart donkeys. Differences in population density and regular attendance at busy markets may mean cart donkeys are generally more habituated to human contact than pack donkeys. Moreover, these differences in the local environment, such as feed availability, social contact, habituation to people, hazards such as vehicles and exhaustion can significantly impact the welfare of donkeys and their resulting behaviour (Geiger & Hovorka 2015).

### Skin lesions

The harnessing typically implemented in cart donkeys sees the entire weight of the cart taken by the donkey’s neck via the ventral neck and cross shaft which is secured using flexible local materials, such as feed bags or elastic cords. Ideally, harnessing would ensure the shoulders and haunches took the weight, with the base of the neck and the chest providing the pulling power (Rodrigues *et al.* 2021). Further, the cart currently in use does not allow for load balancing to optimally decrease the vertical weight carried by the donkeys. Also, there is no way of stopping or slowing the cart through any pressure on the haunches or ‘britching’. The skin lesions seen on 30% (20/66) of the cart donkeys indicates the major welfare concerns associated with this method of harnessing in cart donkeys (Figure 2). Improper harnessing is unfortunately commonplace in developing countries with lack of information and/or resources resulting in abrasive harnessing material and poor weight distribution of the cart (Davis 2019). The presence of sores is variable in other populations of working equids in low- to middle-income countries. Observed rates range from 100% of working donkeys in Pakistan showing healed and open wounds on their front limbs to 16% of working equids in Mexico. Further causes of skin lesions include mistreatment and general overwork (McLean *et al.* 2012; Geiger & Hovorka 2015; Ali *et al.* 2016; Haddy *et al.* 2020; Rodrigues *et al.* 2021).

### Practice-induced conditions (mutilation, hobbling, firing)

Approximately half the donkeys observed had healed wounds on their ears. These injuries are used for identification and to indicate ownership and likely take place due to the practice of free-grazing that could easily result in a donkey roaming far afield. Hobbling injuries were seen in most donkeys with many of these injuries having healed (Sommerville 2018). The prevalence of hobbling varies region-by-region, with 8% of equids surveyed in Mexico (Haddy *et al.* 2020) and 93.4% of 381 donkeys in Southern Ethiopia (Mekuria & Abebe 2010) showing physical signs of tethering. Hobbling entails thin, abrasive ropes being applied with knots that tighten when pressure is applied, and its widespread use here meant the prevalence of hobbling wounds was not surprising. An opportunity for education is presented regarding appropriate materials for use with hobbling, as well as the type of knot that can be employed, i.e. that does not tighten on the donkey’s limb.

Lameness at the walk was not a common finding, with only 6% of the donkeys that pull carts being visibly lame. Of the donkeys that were lame, the owners had noted the condition was present and reported less usage of these donkeys for work. An assessment of lameness prevalence in Kenyan donkeys has not been carried out, although a prevalence of 27% was recorded in Ethiopian working donkeys (Ali *et al.* 2016). In this study, 75% of donkeys were lame in one limb, with those bearing loads in excess of 700 kg showing a greater tendency towards lameness (Ali *et al.* 2016). Fifteen percent of equids were found to be lame in studies in Mexico (Haddy *et al.* 2020) and Mali (McLean *et al.* 2012). A study in Pakistan identified a consistent, pronounced abnormality impeding forward motion at every stride in 42% of the working donkeys assessed (Reix *et al.* 2014). The lower rate of lameness seen in our study may be due to the cart donkeys’ workload (weight of cart, distance travelled, consecutive days worked) being reduced in comparison to the studies mentioned, since the donkeys in the Ethiopian study were pulling carts 6–7 days a week (Ali *et al.* 2016). The methods of assessment and scoring lameness may be stricter in other studies since low-income, food-insecure countries, such as Afghanistan, Egypt, Ethiopia, India, Pakistan and The Gambia have shown a clear prevalence of gait abnormalities and lameness up to 90–100% of animals assessed (Reix *et al.* 2014; Ali *et al.* 2016).

### Healthcare practices of cart and donkey owners

De-worming reportedly differed between groups, with a much higher proportion of cart donkey owners reporting that they administered anthelminthics compared to pack donkey owners. This may be a result of education and awareness of the necessity of de-worming being higher in the cart donkey group. The more arid habitat of the pack donkey perhaps sees a reduced lower intestinal parasitic load enabling donkeys not de-wormed to survive. Comparisons of faecal egg counts between cart and pack donkeys would provide information on the requirement for de-worming in both groups. Owners were offered oral dosing of de-wormer paste for their donkey on completion of the survey and welfare assessment and all accepted. Ivermectin, commonly used in Meru County, was provided and administration was readily tolerated by the donkeys. Paper pamphlets, written in English, describing the de-wormer were offered to the owners to take with them, post-administration.

Owner-reported instances of donkey illness were higher in cart donkeys compared to pack donkeys. A reflection, perhaps of pack donkey owners’ lower level of education as regards signs of illness; owner awareness of disease having been identified as a critical factor in the care of donkeys (Rickards & Toribio 2021). Other possibilities for the differences in the answers include interpretation of the question by owners or actual lower illness rates in the pack donkeys. This difference highlights the need for further investigation into disease occurrence and recognition by owners.

### Reported challenges from donkey owners

This open-ended question allowed owners to highlight the hardest parts of donkey ownership. Despite there being no statistical difference between the groups, pack donkey owners reported the main challenge being feeding and watering their donkeys (81%). This may be a reflection of an overall scarcity of food in the more arid areas these pack donkeys inhabit. Despite donkey slaughter being recognised as a significant welfare issue in Kenya, safety issues, such as slaughter and lost donkeys, were listed as a challenge in 28% of replies. This contrasts with a previous survey by Gichere et al. (2020) which reported theft as the greatest challenge reported by Kenyan donkey owners in Kinrinyaga County (The Brooke 2019; Gichure *et al.* 2020). The decreased concern over donkey theft may reflect the closure of the slaughterhouses in 2020 and banning of commercial donkey slaughter, with a resultant reduction in stolen donkeys. The residual effects of donkey slaughter still exist with the replacement cost of donkeys increasing by 50–100% during the legal period of donkey slaughter from 2012 to 2020 (The Brooke 2019).

### Animal welfare implications

Working donkeys’ welfare is often overlooked due to their low value and social status (Davis 2019). To our knowledge, this is the first study to assess working donkey welfare in Meru County, Kenya. Significant animal welfare concerns were recorded, in both health parameters (low BCS, skin wounds from harnessing, hobbling, and mutilations) and interactions with humans (low-interest and avoidance/aggressive behaviour).

The donkey owners, most notably regarding pack donkeys, experienced insecurity surrounding availability of human and donkey food. The extensive management system makes donkeys very vulnerable to changing climatic conditions with decreased rainfall and feed availability. Improving food resources for donkeys is a very difficult issue to tackle, especially with the overarching human food insecurity in this region.

Non-Governmental Organisations (NGOs) are often present in developing countries with a role addressing welfare issues facing working equids. Ensuring each region provides information on welfare improvements carried out gives greater capacity for positive change (Norris *et al.* 2021). It is a challenge to quantify the population of donkeys working in each region of Kenya since most livestock censuses do not include donkeys. This study describes welfare and owner challenges in Meru County, Kenya, to provide a benchmark for interventions including improved harnessing and cart design.

The reduction of negative affective states and increase in positive states are goals for the improvement of animal welfare (Mellor 2016). The promotion of positive affective states for these working animals could stem from appropriate handling that is reflective of normal donkey behaviour and responses. Improvements in harnessing and cart design could potentially lower the fatigue experienced by donkeys, and decrease the negative interactions from humans, such as prodding and whipping, when the speed of work decreases in the donkeys. Owner/driver education regarding behavioural signs of fatigue in donkeys is recommended, including a decline in speed of working, unwillingness to continue, inco-ordination and excitement after work (Bukhari & Parkes 2023). Training and equipment to enable donkeys to be moved around using halters or bridles, rather than hobbles or poking/prodding would likely improve human-donkey interactions as well decrease the prevalence of skin wounds.

The results of this research provide important foundational information that can be used for future research aiming to improve the welfare of these donkeys.

## Conclusion

This study describes the baseline health and welfare as well as husbandry and working conditions of a subset of pack and cart donkeys in Meru County, Kenya. Significant welfare concerns, such low BCS, presence of skin wounds in the cart donkeys, hobbling and mutilation wounds were identified. Significant differences between the cart and pack donkeys were noted in their signalment, BCS, skin lesions, as well as chin contact and tail tuck. This could reflect differences in work type, food availability, and owner handling. Numbers of donkeys owned, de-worming regularity, and rate of reported illness differed significantly between the cart and pack donkeys. Level of owner education and work type may be factors in these differences. This initial survey will serve as a benchmark for the planned introduction of various interventions, including modified carts and health clinics.

## Supporting information

Mellish and Stull supplementary material Mellish and Stull supplementary material

## Supplementary material

The supplementary material for this article can be found at http://doi.org/10.1017/awf.2025.10031.
