# Supplementary material for: Working donkey welfare assessment and owner survey in Meru County, Kenya
Source: Anim Welf. 2025 Aug 22;34:e60. doi: 10.1017/awf.2025.10031 (PMC12451387; doi:10.1017/awf.2025.10031)
Supplement: Mellish and Stull supplementary material [file S0962728625100316sup001.pdf]

Working donkey welfare assessment and owner survey in Meru County, Kenya

Martha A Mellishhttps://orcid.org/0000-0002-0557-8384 and Jason W Stull https://orcid.org/0000-0002-9028-8153

Department of Health Management, Atlantic Veterinary College, University of Prince Edward Island, PE, Canada C0A 1T0

Author for correspondence: Martha A Mellish, email: mmellish@upei.ca

Supplementary material

Full results of SEBWAT

Question (n)	Transport Good by Cart, TGC (%)	Transport Good by Pack, TGP (%)	P-value
Sex (TGC=68; TGP=33)			<0.001[a]
Male	68 (100%)	7 (21%)	
Female	0	26 (79%)	
Age (TGC=66; TGP=34)			0.45[a]
Less than 3.5 years	5 (8%)	6 (18%)	
3.5-7.9 years	21 (32%)	8 (24%)	
8.0-12.0 years	19 (29%)	10 (29%)	
Over 12 years	21 (32%)	10 (29%)	
Height (hands) (TGC=64; TGP=33)			0.002[b]
Mean (SD)	10.1 (0.4)	9.9 (0.4)	
Median (range)	10.1 (9, 11.1)	10.1 (9.1, 10.2)	
Weight via tape; kgs(TGC=63; TGP=33)			
Mean (SD)	140.5 (19.5)	133.6 (22.7)	
Median (range)	143.2 (99.1, 190.9)	136.4 (110, 186.8)	
Body Condition (TGC=66; TGP=32)			<0.0001[b]
Mean (SD)	2.3 (0.5)	1.6 (0.5)	
Median (range)	2 (1, 3.5)	1.5 (1, 2.5)	
Observer Approach (TGC=68; TGP=34)			0.7[c]
Score 0	4 (6%)	1 (3%)	
Score 1	43 (63%)	20 (59%)	
Score 2	21 (31%)	13 (38%)	
Chin Contact (head shy) (TGC=68; TGP=34)			<0.001[a]
0 for chin contact	30 (44%)	1 (3%)	
1 for chin contact	38 (56%)	33 (97%)	
Tail Tuck (TGC=68; TGP=34)			<0.001[a]
Score 0	37 (54%)	1 (3%)	
Score 1	31 (46%)	33 (97%)	

General Attitude (TGC=68; TGP=34)			
Score 0	13 (19%)	1 (3%)	0.08[a]
Score 1	43 (63%)	26 (76%)	
Score 2	12 (18%)	7 (21%)	
Spinal Contact (TGC=66; TGP=34)			0.8[c]
Score 0	58 (88%)	29 (85%)	
Score 1	8 (12%)	5 (15%)	
Neck/Severity (TGC=66; TGP=34)			< 0.001[c]
Score 0	46 (70%)	34 (100%)	
Score 1	18 (27%)	0	
Score 2	2 (3%)	0	
Neck/Size (TGC=50; TGP=34)			0.1[c]
Score 0	43 (86%)	34 (100%)	
Score 1	4 (8%)	0	
Score 2	2 (4%)	0	
Score 3	1 (2%)	0	
Breast & Shoulder/Severity (TGC=68; TGP=34)			0.09[c]
Score 0	60 (88%)	34 (100%)	
Score 1	7 (10%)	0	
Score 2	1 (1%)	0	
Breast & Shoulder/Size (TGC=68; TGP=34)			0.5[c]
Score 0	63 (93%)	34 (100%)	
Score 1	2 (3%)	0	
Score 2	3 (4%)	0	
Forelimbs/Severity (TGC=68; TGP=34)			0.1[c]
Score 0	59 (87%)	34 (100%)	
Score 1	6 (9%)	0	
Score 2	3 (4%)	0	
Forelimbs/ Size (TGC=68; TGP=34)			0.3[c]
Score 0	62 (91%)	34 (100%)	
Score 1	4 (6%)	0	
Score 2	2 (3%)	0	

Withers & Spine/Severity (TGC=68; TGP=34)			0.1[c]
Score 0	56 (82%)	33 (97%)	
Score 1	8 (12%)	1 (3%)	
Score 2	4 (6%)	0	
Withers & Spine/Size (TGC=68; TGP=34)			0.09[c]
Score 0	61 (90%)	34 (100%)	
Score 1	7 (10%)	0	
Ribs & Flank/Severity (TGC=67; TGP=34)			0.006[c]
Score 0	66 (99%)	29 (85%)	
Score 1	0	4 (12%)	
Score 2	1 (1%)	1 (3%)	
Ribs & Flank/Size (TGC=67; TGP=34)			0.04[c]
Score 0	66 (99%)	30 (88%)	
Score 1	1 (1%)	3 (9%)	
Score 2	0	1 (3%)	
Mutilations: Tail, Ear, Muzzle (TGC=68; TGP=34)			0.5[a]
Score 0	37 (54%)	16 (47%)	
Score 1	31/68 (46%)	18/34 (53%)	
Firing lesion: Body Areas (TGC=68; TGP=34)			0.3[c]
Score 0	58 (85%)	33 (97)	
Score 1	5 (7%)	1 (3%)	
Score 2	5 (7%)	0	
Firing lesion: Severity (TGC=68; TGP=34)			0.2[c]
Score 0	59 (87%)	33 (97%)	
Score 1	9 (13%)	1 (3%)	
Hobbling lesion: Body areas (TGC=68; TGP=34)			0.1[a]
Score 0	14 (21%)	7 (21%)	
Score 1	12 (18%)	1 (3%)	
Score 2	42/68 (62%)	26/34 (76%)	
Hobbling lesion: Severity (TGC=68; TGP=34)			0.03[c]
Score 0	14 (21%)	7 (21%)	
Score 1	53/68 (78%)	22/34 (65%)	

Score 2	1 (1%)	5 (15%)	
Gait (TGC=68; TGP=34)			0.7[c]
Score 0	64 (94%)	34 (100%)	
Score 1	1 (1%)	0	
Score 2	3 (4%)	0	
Lower limb swelling Fore (TGC=68; TGP=34)			1[c]
Score 0	65 (96%)	33 (97%)	
Score 1	2 (3%)	1 (3%)	
Score 2	1 (1%)	0	
Lower limb swelling Hind (TGC=68; TGP=34)			0.1[c]
Score 0	64 (94%)	28 (82%)	
Score 1	2 (3%)	4 (12%)	
Score 2	2 (3%)	2 (6%)	

Columns may not sum to 100% due to rounding.

[a] Pearson chi square test

[b] Two-sample Wilcoxon rank-sum (Mann–Whitney) test

[c] Fisher's exact test

Survey questions

How many donkeys do you or your family own or drive?
What feed is provided for the donkeys?
How do you get the donkey feed?

Harvest yourself
Buy
Graze on common property
Other

Do you vaccinate your donkey?
If you do vaccinate, for which diseases?

Do you de-worm your donkey?
If yes, how many times per year?
If yes, how what do you use to de-worm?

Do they have their hooves trimmed?
If so, how many times per year?

How many days a week do the donkeys work?

How much weight do the donkeys carry on average?

When the donkeys work:
For how many hours are they walking?
For how many hours are they standing with weight on their back?
Have you owned a donkey that has been sick?

If yes, do you ask anyone for medical treatment when your donkey is sick?
If yes, who do you ask for advice or medicine?
Veterinarian
Veterinary technician
Other donkey owners
Other
If you don’t ask for treatment (no), why not?
Financial reasons
No one available
No medication available
Other
Where did you get your donkey?
What are the challenges you face with your donkey?

Table 6: Responses to donkey owners surveys, for those donkeys that transport goods by cart (n=28) and by pack (n=30), Kenya

Question (n)	Transport Good by Cart (%)	Transport Good by Pack (%)	P-value
How many donkeys do you or your family own or drive? (TGC=28; TGP=30)			
Mean (SD)	2.4 (1.1)	1.6 (0.9)	0.0001[a]
Median (range)	2 (1-6)	1 (1-4)	
What feed is provided for the donkeys? (TGC=22; TGP=25)			NP
Grass	14 (64%)	8 (32%)	
Weeds	9 (41%)	4 (16%)	

Concentrates	1 (5%)	0	
Maize	3 (14%)	3 (12%)	
Beans	0	1 (4%)	
Nursing	0	1 (4%)	
Milk	0	1 (4%)	
Wheat	1 (5%)	0	
Napier grass	4 (18%)	0	
Hay	1 (5%)	0	
Bran	4 (18%)	0	
Fruit	1 (5%)	0	
How do you get the donkey feed? (TGC=28; TGP=25)			NP
Harvest yourself	9 (32%)	5 (20%)	
Buy	1 (4%)	2 (8%)	
Graze on common property	26 (93%)	25 (100%)	
Do you vaccinate your donkey? (TGC=27; TGP=30)			0.1[b]
No	22 (81%)	29 (97%)	
Yes	1 (4%)	0	
Yes, 7-10 years ago	4 (15%)	1 (3%)	
Do you de-worm your donkey? (TGC=20; TGP=30)			<0.001[c]
No	1 (5%)	25 (83%)	
Yes	19 (95%)	5 (17%)	
If yes, how many times per year?		?	
Every 3 months	10	1	
Less frequently than every 3 months	8	6	
How many days a week do the donkeys work? (TGC=21; TGP=29)			<0.001[b]
1-3 days a week	16 (76%)	4 (14%)	
4-7 days a week	5 (24%)	22 (76%)	
Not working (too young, too old, lame)	0	3 (10%)	
How much weight do the donkeys carry on average? (TGC=26; TGP=28)			
80 kg or less	0	21 (75%)	
More than 80 kg	26 (100%)	7 (25%)	
Mean (SD)	461.9 (197.3)	83.9 (47.1)	<0.0001[a]
Median (range)	500 (120-900)	80 (20-300)	

When the donkeys work, for how many hours are they walking? (TGC=24; TGP=26)			0.018[b]
Less than 1 hour	2 (8%)	1 (4%)	
1-1.99 hours	4 (17%)	8 (31%)	
2-2.99 hours	4 (17%)	12 (46%)	
3-3.99 hours	4 (17%)	3 (12%)	
Greater than 4 hours	10 (42%)	2 (8%)	
Have you owned a donkey that has been sick? (TGC=27; TGP=24)			0.001[c]
Yes	22 (81%)	9 (38%)	
No	5 (19%)	15 (63%)	
If yes, reporting pneumonia, coughing, common cold, sneezing	6	3	
If yes, reporting tetanus	5	1	
If yes, reporting foot problems	2	1	
If yes, reporting Other (dull, sluggish, long hair, worms, no specific cause, abortion, sick after birth)	6	2	
What are the challenges you face with your donkey? (TGC=26; TGP=27)			
feeding (food+water+fodder+feed)	4 (15%)	22 (81%)	
Behaviour (slow, behaviour, tired, rest, refuse)	7 (27%)	2 (7%)	
Health (de-worming, worms, disease, wounds, sick, disease, foot, limp, pneumonia, tsetse fly)	16 (62%)	11 (41%)	
Safety (falling, slaughter, predators, tether, lost, traffic, park, cut)	9 (35%)	6 (22%)	

[a] Two-sample Wilcoxon rank-sum (Mann–Whitney) test

[b] Fisher's exact test

[c] Pearson chi square test

NP = Not performed

TGC=Transport Goods by Cart

TGP=Transport Goods by Pack
